# Association of neuroticism with incident dementia, neuroimaging outcomes, and cognitive function

**DOI:** 10.1002/alz.14071

**Published:** 2024-07-10

**Authors:** Yaqing Gao, Najaf Amin, Cornelia van Duijn, Thomas J Littlejohns

**Affiliations:** ^1^ Nuffield Department of Population Health University of Oxford Oxford UK

**Keywords:** apolipoprotein E, cognitive function, dementia, genetics, longitudinal, mediation, neuroimaging, neuroticism, polygenic risk, UK Biobank

## Abstract

**INTRODUCTION:**

Higher neuroticism might be associated with dementia risk. Here we investigated modification by genetic predisposition to dementia, mediation by mental health and vascular conditions, neuroimaging outcomes, and cognitive function.

**METHODS:**

Cox proportional‐hazards models were used to assess the association between neuroticism score and incident dementia over up to 15 years in 1,74,164 participants. Cross‐sectional analyses on dementia‐related neuroimaging outcomes and cognitive function were conducted in 39,459 dementia‐free participants.

**RESULTS:**

Higher neuroticism was associated with an 11% higher risk of incident dementia, especially vascular dementia (15% higher risk), regardless of genetic predisposition to dementia. Mental and vascular conditions mediated the association of neuroticism with all‐cause dementia and vascular dementia. Neuroticism was associated with higher cerebrovascular pathology, lower gray matter volume, and worse function across multiple cognitive domains.

**DISCUSSION:**

Neuroticism could represent a risk factor for dementia, and vascular and mental health might drive these associations.

**Highlights:**

- Neuroticism was associated with an increased risk of incident all‐cause dementia, particularly vascular dementia.
- Associations were not modified by genetic predisposition to dementia.
- Associations were largely mediated by mental and vascular conditions.
- Neuroticism was associated with increased cerebrovascular pathology and lower gray matter volume.
- Neuroticism was associated with worse function across multiple cognitive domains.

## 1 BACKGROUND

Neuroticism is a personality trait comprising components such as irritability, worry, self‐consciousness, loneliness, and vulnerability.^1^ It stabilizes by adolescence and remains relatively consistent across the life course.^2^ Neuroticism is measured using a continuous scale, with higher scores indicating greater neuroticism. Neuroticism has been linked to a greater risk of adverse health outcomes and has been identified as a potential risk factor for dementia. In 2021, a meta‐analysis of 12 longitudinal studies, totaling 33,054 participants (1806 dementia cases), found that increased neuroticism was associated with a greater risk of developing dementia.^3^ However, most of these studies have short follow‐up periods (<10 years), which makes it difficult to draw inferences on whether neuroticism is a prodromal symptom or a cause of dementia. Therefore, it is important to investigate the association in population‐based cohorts with longer follow‐up periods.

Furthermore, previous studies have investigated the direct association between neuroticism and dementia; however, neuroticism might indirectly increase dementia risk via other modifiable factors. This is particularly important when considering preventative approaches, as there is limited evidence that neuroticism can be modified by behavioral interventions.^4^ Prospective studies have linked higher neuroticism with an increased risk of subsequent depression,^5^ anxiety disorders,^5^ cardiovascular diseases (CVDs),^6^ and hypertension.^7^ In addition, Mendelian randomization studies show evidence for a unidirectional, but not bi‐directional, causal influence of neuroticism on these conditions.^8^, ^9^, ^10^ Mental and vascular health conditions might represent viable targets in individuals with higher neuroticism, especially as these are well‐recognized risk factors for dementia and neuroticism is unlikely to be easily modified.^11^ However, there is a lack of studies investigating whether the link between neuroticism and dementia risk is mediated through these conditions. Moreover, the preclinical stage of dementia is marked by subtle changes in brain health, including structural brain changes, cerebrovascular pathology, and cognitive decline.^12^ Understanding whether neuroticism is associated with adverse brain health could provide insights into mechanisms underlying its association with dementia.

**RESEARCH IN CONTEXT**

1. **Systematic review**: Literature reviews via PubMed suggest that neuroticism is associated with an increased risk of developing mental health and vascular conditions, which are well‐established risk factors for dementia. However, studies on the association between neuroticism and dementia are limited by small numbers of dementia cases and short follow‐up periods. In addition, less is known about the potential interaction between neuroticism and genetic risk for dementia, whether mental health and vascular conditions mediate the relationship, or the association of neuroticism with specific cognitive domains and neuroimaging outcomes.
2. **Interpretation**: In 1,74,164 participants followed up to 15 years (N ≈ 6000 incident dementia cases), higher neuroticism was associated with an increased risk of all‐cause dementia, with a stronger association observed for vascular dementia. The associations were not modified by genetic predisposition to dementia. We found that the associations were largely mediated by mental and vascular conditions. Neuroticism was also associated with evidence of increased cerebrovascular pathology, lower gray matter volume, and worse function across multiple cognitive domains.
3. **Future directions**: Future studies should examine the relationship of neuroticism with physical conditions, neuroimaging outcomes, and cognitive function longitudinally. Exploring molecular and neurological pathways linking neuroticism and dementia could further inform intervention targets.

In the current study, we first investigated the association between neuroticism and the risk of incident dementia in a large population‐based cohort followed for up to 15 years. Next, we assessed whether and how much this association is mediated through mental and vascular conditions. We also examined mediation by a metabolic condition (diabetes), a well‐established dementia risk factor but lacking evidence of a link to neuroticism. It serves as a negative control, and we expected its mediation effect to be smaller than that of mental and vascular conditions, or non‐significant. Finally, we explored the association with neuroimaging and cognitive outcomes.

## 2 METHODS

### 2.1 Study population

UK Biobank (UKB) is a population‐based cohort study that recruited more than 5,00,000 individuals 40–69 years of age from England, Scotland, and Wales between 2006 and 2010.^13^ Baseline assessments were conducted using touchscreen questionnaires, verbal interviews, and physical measurements to collect sociodemographic, lifestyle, and health‐related data. Blood samples were collected for additional assays, including genotyping. Follow‐up data were obtained by cohort‐wide linkage to electronic health records.

**FIGURE 1:**
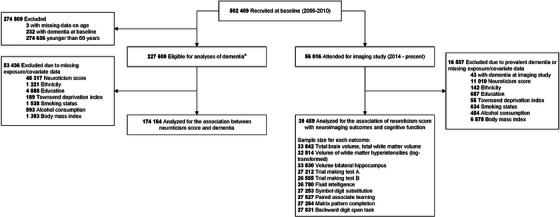
Flow diagram of analyses. ^a^Sensitivity analysis was conducted in this population using multiple imputation to account for missing data on the exposure and covariates.

We excluded participants who were younger than 60 years of age at baseline (*n* = 2,74,636) to ensure that the analytic sample was restricted to those at high risk of developing late‐onset dementia (typically defined as onset after age 65) during the 15‐year follow‐up period. We further excluded participants with self‐reported prevalent cognitive impairment or dementia or hospital‐diagnosed dementia at baseline (*n* = 232), missing neuroticism (*n* = 48,317), or covariate (*n* = 7992) data. Consequently, the final sample size consisted of 1,74,164 participants (Figure 1). Characteristics of those with and without missing neuroticism data are provided in eTable 1.

Neuroimaging and cognitive function analyses were conducted on a subset of the participants from the baseline cohort invited to join the UKB imaging study, starting from 2014.^14^ During the imaging visit, participants underwent brain magnetic resonance imaging (MRI) using a 3 Tesla Siemens Skyra scanner (software VD13) and a standard Siemens 32‐channel head coil as well as a repeat of all baseline measures. Brain scans underwent standardized processing and quality control procedures to generate imaging‐derived phenotypes (IDPs).^15^ Cognitive assessments were administered through a touchscreen questionnaire.^16^ Of 56,016 participants with IDPs available, exclusions were made for those with prior diagnoses of cognitive impairment or dementia (*n* = 43), as well as those with missing neuroticism (*n* = 11,010) or covariate data (*n* = 4372). Because we were interested in investigating the association between neuroticism and brain health outcomes in dementia‐free individuals, we did not exclude based on age. This resulted in a final sample size of 39,459 participants.

UKB received ethical approval from the North West Multi‐centre Research Ethics Committee (MREC) and all participants provided informed consent via electronic signature through the touchscreen.

### 2.2 Assessment of neuroticism

Neuroticism was assessed using the Eysenck Personality Questionnaire‐Revised Short Form (EPQ‐RS).^17^ The EPQ‐RS shows high reliability (Cronbach's alpha 0.80–0.84)^17^ and correlates strongly (*r* = 0.85)^18^ with neuroticism score on the neuroticism‐extraversion‐openness (NEO) Five‐Factor Inventory, another well‐established measurement tool. Its psychometric properties remain consistent across cultures and age groups.^19^, ^20^ The EPQ‐RS consists of 12 items, with each item measuring a single neurotic trait (eTable 2). Response options were “No” and “Yes,” which were recoded as 0 and 1, respectively. An overall neuroticism score ranging from 0 to 12 was derived by summing the responses to all items. A higher score indicates higher levels of neuroticism. Finally, the neuroticism score was standardized to have a mean of zero and a standard deviation (SD) of one to create a neuroticism z‐score. Neuroticism measured through the touchscreen questionnaire at baseline was used as the exposure in the dementia analyses, and neuroticism measured through the touchscreen questionnaire at the imaging assessment was used as the exposure in the neuroimaging and cognitive function analyses.

### 2.3 Incident dementia outcomes

Incident dementia was defined as the first hospital inpatient primary or secondary diagnosis of dementia or dementia as an underlying or contributory cause of death. The sensitivity and specificity for identifying all‐cause dementia using England hospital inpatient data are 78.0% and 92.0%,^21^ respectively, with potential further improvement through the use of death register data. Both hospital inpatient data and death registers employed the International Classification of Diseases (ICD) coding system to encode diagnostic information. The ICD code list for dementia and its subtypes was developed and validated by the UK Biobank Outcome Adjudication group (see eTable 3 for list of ICD codes used to define dementia and its subtypes).^22^

### 2.4 Neuroimaging and cognitive outcomes

IDPs were derived from T1‐weighted and T2‐weighted fluid‐attenuated inversion recovery (T2‐FLAIR) structural images. T1 scans enabled the measurement of brain tissue and structure volumes, whereas T2‐FLAIR scans facilitated the identification of pathological changes such as chronic plasma leakage and demyelination.^14^ Selected imaging markers to indicate brain health included total brain volume, total white matter volume, total gray matter volume, volume of bilateral hippocampus, and volume of white matter hyperintensities (WMHs). WMHs were log‐transformed due to their skewed distribution. eFigure 1 shows the raw and transformed distribution of the imaging outcomes.

IDPs were corrected for image‐related confounds following published guidelines.^23^ Confounds included head size (based on the volumetric scaling from the T1 head image to standard atlas), head motion, head position (X, Y, Z brain center of gravity, and table position), and imaging center (eTable 4). We fit a linear regression model with IDPs as the outcome and image‐related confounds as predictors. The scaled residuals from this regression served as the outcomes in the neuroimaging analysis.

We selected cognitive tests with demonstrated reliability and validity and covering different domains,^16^ including executive function (Trail Making Test Parts A and B), verbal and numerical reasoning (fluid intelligence), working memory (backward digit span task), complex processing speed (symbol digit substitution), verbal declarative memory (paired associate learning), and non‐verbal reasoning (matrix pattern completion). Performance scores for each task were summarized in eTable 4. Raw scores for all tests were standardized as z‐scores within 5‐year age bands, with higher z‐scores indicating better performance.

### 2.5 Covariates and mediators

We selected covariates based on established associations with dementia risk, including sociodemographic and lifestyle factors. The following covariates were measured at baseline: sex (female and male), ethnicity (White and non‐White), and Townsend deprivation index (TDI, in quintiles). TDI is a summary measure of material deprivation that takes into account factors such as unemployment, overcrowding, non‐car ownership, and non‐home ownership.^24^ Age, education (primary, secondary, post‐secondary non‐tertiary, and tertiary), smoking status (never, previous, and current smoker), alcohol consumption (≤4 times/week, daily, or almost daily), and body mass index (BMI) (normal [<25 kg/m^2^], overweight [25–30 kg/m^2^], and obese [≥30 kg/m^2^]) were measured both at baseline (adjusted for in the dementia analyses) and at the imaging assessment (adjusted for in the neuroimaging and cognitive function analyses). eTable 4 provides detailed information on measurement, definition, and classification of the covariates.

We considered baseline history of depression, anxiety and stress‐related disorders, ischemic heart disease (IHD), hypertension, and diabetes as potential mediators in the association between neuroticism and dementia. Disease histories were obtained from two sources: self‐reported medical conditions obtained during the verbal interview at baseline and hospital diagnoses of diseases prior to the baseline visit (eTable 3 and eTable 4).

### 2.6 Statistical analysis

#### 2.6.1 Association of neuroticism with all‐cause dementia

Cox proportional‐hazards regression was used to test the association between neuroticism z‐score and incident dementia. In a separate model, neuroticism score was categorized into 12 groups, with each point representing a separate category and “0” (lowest neuroticism level) as the reference group. The analysis was adjusted for covariates measured at baseline, with follow‐up time as the timescale. Participants were followed from the date of attending baseline assessment until a record of dementia diagnosis/cause of death, death, or the censoring date of hospital inpatient records (October 31, 2022, for England, August 31, 2022 for Scotland, and May 31, 2022 for Wales), whichever occurred first. Violation of the proportional‐hazards assumption was assessed visually using Schoenfeld's residuals. We performed sensitivity analyses, using multiple imputation to account for missing data (eMethods), and including all UKB participants without dementia in the analysis with no restrictions on baseline age. To investigate potential reverse causation due to preclinical dementia affecting exposure status prior to a dementia diagnosis, the main analysis was repeated, restricting to three separate follow‐up periods: ≤5 years, >5 to ≤10 years, and >10 years.

#### 2.6.2 Effect modification by genetic risk factors

We investigated the effect modification by genetic risk for dementia. We measured genetic risk using apolipoprotein E (*APOE*) genotypes and a non‐*APOE* polygenic risk score (PRS) for Alzheimer's disease (AD) (hereafter referred to as “non‐*APOE* PRS”). Details of how these genetic risk factors were derived are provided in eMethods. We incorporated a two‐way interaction term between neuroticism z‐score and the genetic risk factors and examined significant interaction effects using the likelihood ratio test. We then stratified the analyses by *APOE* genotypes (carrier of *APOE* ε2, *APOE* ε3/ε3, or *APOE* ε4) and non‐*APOE* PRS status (quintile groups). Only individuals who self‐reported as being of a White ethnic background were included in the analyses stratified by non‐*APOE* PRS status.

#### 2.6.3 Mediation analysis

Using a marginal structural model approach within the counterfactual framework (eMethods),^25^ we decomposed the total effect of neuroticism on dementia into natural direct effects and natural indirect effects. The natural direct effect represents the effect of neuroticism on dementia through pathways unrelated to the specific disease's history, whereas the natural indirect effect represents the mediating effect of the association between neuroticism and dementia through the disease. The proportion mediated via the disease was calculated by dividing the natural indirect effect by the total effect.

#### 2.6.4 Association of neuroticism with neuroimaging outcomes and cognitive function

The association of the neuroticism z‐score (measured at the imaging assessment) with neuroimaging outcomes and cognitive function was tested using linear regression. Covariates such as sex, ethnicity, and TDI measured at baseline were included, and for other covariates (age, education, smoking status, alcohol consumption, and BMI), measurements at the imaging assessment were used. Furthermore, we performed analyses stratified by age group.

## 3 RESULTS

### 3.1 Association of neuroticism with dementia and its subtypes

The final sample included 1,74,164 participants (Figure 1). During a median follow‐up of 13.5 years (interquartile range [IQR] 12.5–14.0), 5974 participants developed incident all‐cause dementia, of which 2741 consisted of AD and 1364 consisted of vascular dementia (VaD). Compared with those with lower neuroticism scores, individuals with higher neuroticism scores were more likely to be female, have lower socioeconomic status, be current or previous smokers, and have a history of depression, anxiety, CVD, and diabetes (Table 1). Levels of neuroticism were comparable across *APOE* genotypes and non‐*APOE* PRS levels. Participants who developed dementia during follow‐up showed a risk profile similar to that for people with higher neuroticism levels regarding socioeconomic status and comorbidities but were more likely to be male, *APOE* ε4 carriers, and have a high non‐*APOE* PRS (eTable 5).

**TABLE 1:** Baseline characteristics of participants.

		Neuroticism score tertile		
Characteristic	Overall	1	2	3
*N*	1,74,164	58,055	58,055	58,054
Age, mean (SD)	64.36 (2.96)	64.50 (2.97)	64.39 (2.96)	64.18 (2.95)
Male, *n* (%)	83,849 (48.1)	33,961 (58.5)	26,813 (46.2)	23,075 (39.7)
Townsend deprivation index quintile, *n* (%)				
1 (Least deprived)	38,198 (21.9)	13,416 (23.1)	13,011 (22.4)	11,771 (20.3)
2	38,197 (21.9)	13,146 (22.6)	12,798 (22.0)	12,253 (21.1)
3	36,256 (20.8)	12,263 (21.1)	12,233 (21.1)	11,760 (20.3)
4	32,841 (18.9)	10,578 (18.2)	10,986 (18.9)	11,277 (19.4)
5 (Most deprived)	28,672 (16.5)	8652 (14.9)	9027 (15.5)	10,993 (18.9)
Education, *n* (%)				
Primary	44,741 (25.7)	12,601 (21.7)	14,409 (24.8)	17,731 (30.5)
Secondary	36,128 (20.7)	11,210 (19.3)	12,230 (21.1)	12,688 (21.9)
Post‐secondary non‐tertiary	24,329 (14.0)	8450 (14.6)	8451 (14.6)	7428 (12.8)
Tertiary	68,966 (39.6)	25,794 (44.4)	22,965 (39.6)	20,207 (34.8)
Ethnic group—White, *n* (%)	1,70,011 (97.6)	56,536 (97.4)	56,777 (97.8)	56,698 (97.7)
BMI, *n* (%)				
Normal	51,858 (29.8)	16,464 (28.4)	17,547 (30.2)	17,847 (30.7)
Overweight	78,883 (45.3)	27,283 (47.0)	26,279 (45.3)	25,321 (43.6)
Obese	43,423 (24.9)	14,308 (24.6)	14,229 (24.5)	14,886 (25.6)
Drinking daily or almost daily, *n* (%)	1,32,224 (24.1)	43,209 (25.6)	44,021 (24.2)	44,994 (22.5)
Smoking status, *n* (%)				
Never	86,655 (49.8)	30,194 (52.0)	29,153 (50.2)	27,308 (47.0)
Previous	73,118 (42.0)	23,351 (40.2)	24,320 (41.9)	25,447 (43.8)
Current	14,391 (8.3)	4510 (7.8)	4582 (7.9)	5299 (9.1)
*APOE* genotype, *n* (%)				
*APOE* ε2 carriers	22,236 (12.8)	7388 (12.7)	7333 (12.6)	7515 (12.9)
*APOE* ε3/ε3 carriers	1,00,368 (57.6)	33,492 (57.7)	33,508 (57.7)	33,368 (57.5)
*APOE* ε4 carriers	43,683 (25.1)	14,625 (25.2)	14,569 (25.1)	14,489 (25.0)
Missing/ambiguous genotypes^a^	7877 (4.5)	2550 (4.4)	2645 (4.6)	2682 (4.6)
Non‐*APOE* PRS^b^, *n* (%)				
Low	27,996 (16.1)	9228 (15.9)	9534 (16.4)	9234 (15.9)
Intermediate	84,795 (48.7)	28,349 (48.8)	28,118 (48.4)	28,328 (48.8)
High	28,559 (16.4)	9780 (16.8)	9422 (16.2)	9357 (16.1)
Missing	32,814 (18.8)	10,698 (18.4)	10,981 (18.9)	11,135 (19.2)
Medical history, *n* (%)				
Depression	8744 (5.0)	638 (1.1)	1731 (3.0)	6375 (11.0)
Anxiety and stress disorder	2800 (1.6)	279 (0.5)	522 (0.9)	1999 (3.4)
Hypertension	64,338 (36.9)	20,052 (34.5)	21,331 (36.7)	22,955 (39.5)
Ischemic heart disease	15,176 (8.7)	4600 (7.9)	4889 (8.4)	5687 (9.8)
Diabetes	12,083 (6.9)	3957 (6.8)	3934 (6.8)	4192 (7.2)
Neuroticism z‐score, mean (SD)	0.00 (1.00)	−1.01 (0.21)	−0.17 (0.30)	1.18 (0.63)

Abbreviations: BMI, body mass index; N, number of participants; PRS, polygenic risk score; SD, standard deviation.

^a^ *APOE* ε1/ε3 and *APOE* ε2/ε4 genotypes.

^b^ Low (lowest PRS quintile), intermediate (PRS quintiles 2–4), and high (highest PRS quintile).

**FIGURE 2:**
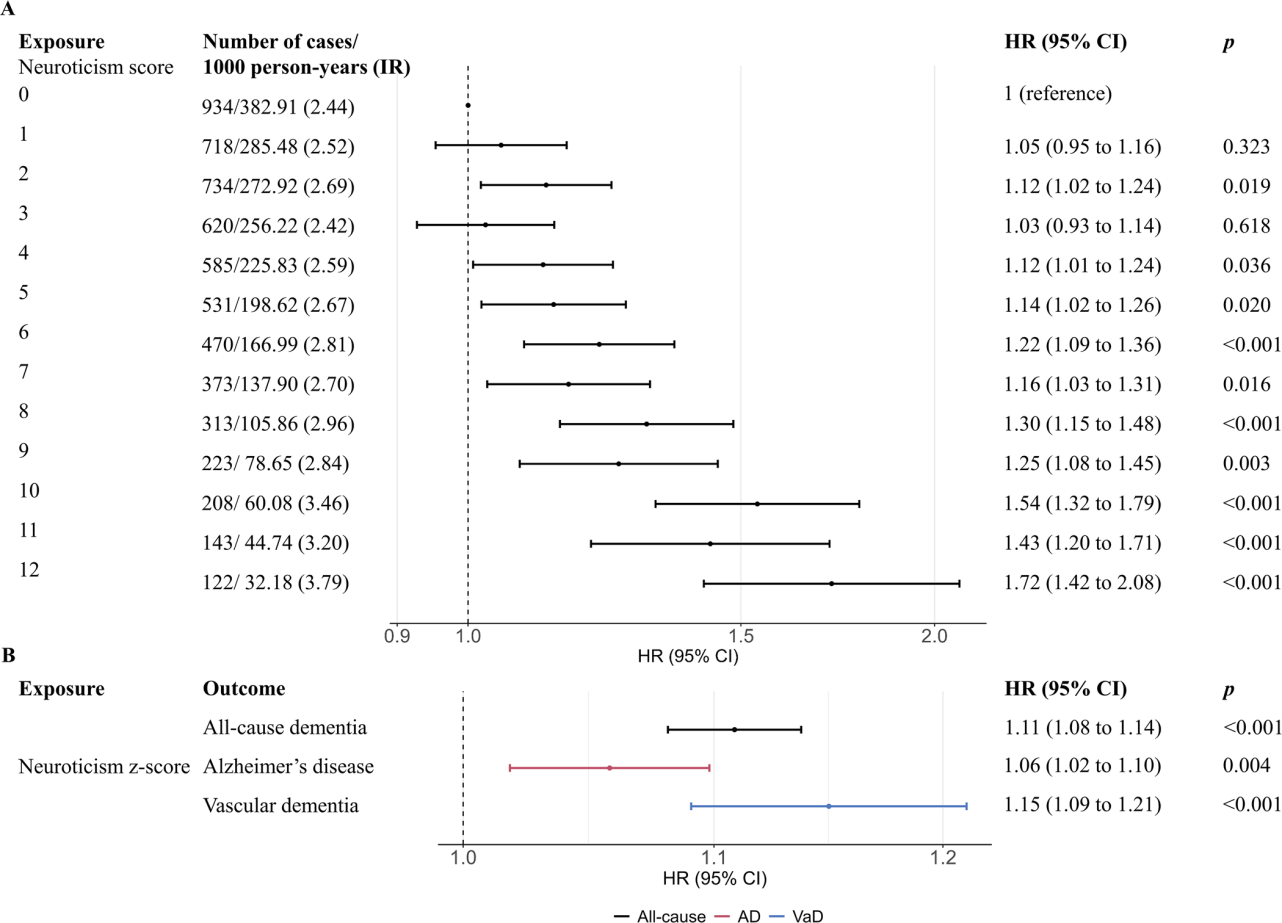
Associations between neuroticism score (original values and z‐score) and incident dementia. (A) Association between neuroticism score and incident all‐cause dementia. (B) Association between neuroticism z‐score and incident all‐cause dementia and subtypes of dementia. Models were adjusted for age, sex, ethnicity, education, quintiles of the Townsend deprivation index (TDI), smoking status, alcohol consumption, and body mass index (BMI). HR, hazard ratio; CI, confidence interval; AD, Alzheimer's disease; VaD, vascular dementia.

There was a dose–response association between neuroticism score and risk of incident dementia (Figure 2A), with a stronger association observed in those who developed VaD. The association with AD was slightly weaker (eFigure 2). A one‐unit increase in neuroticism z‐score was associated with an 11% (hazard ratio [HR] 1.11, 95% confidence interval [CI], 1.08 to 1.14]), 6% (HR 1.06, 95% CI, 1.02 to 1.10), and 15% (HR 1.15, 95% CI, 1.09 to 1.21) higher risk of incident all‐cause dementia, AD, and VaD, respectively (Figure 2B). When restricting to >10 years of follow‐up, the association of neuroticism z‐score with all‐cause dementia and VaD was attenuated but remained significant (eFigure 3). The sensitivity analyses using multiple imputation and removing baseline age restrictions produced results that were similar to the main findings (eTable 6 and eTable 7).

### 3.2 Effect modification by genetic risk factors

**FIGURE 3:**
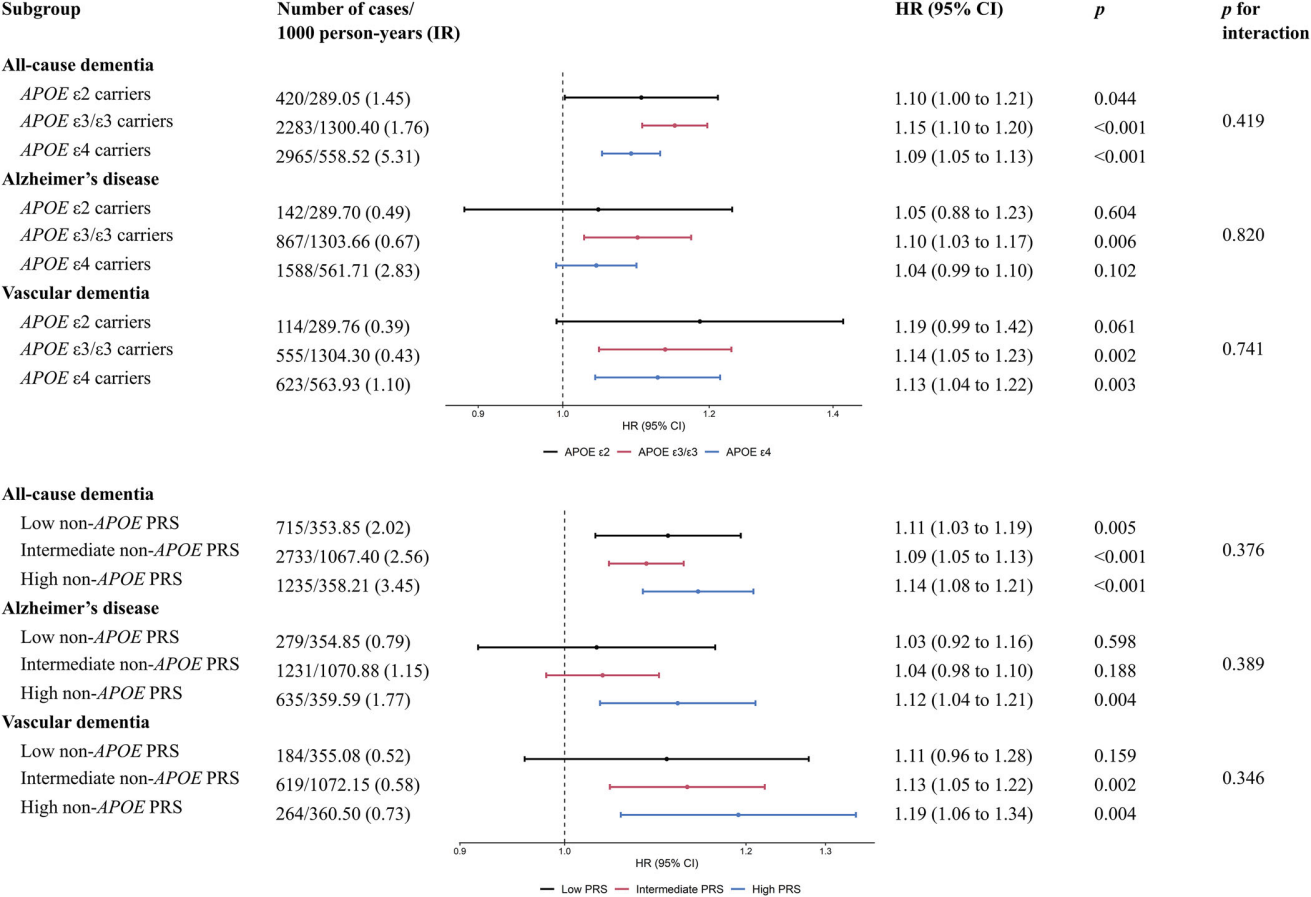
Association between neuroticism z‐score and incident dementia by *APOE* genotype and non‐*APOE* PRS. Models were adjusted for age, sex, ethnicity, education, quintiles of the Townsend deprivation index (TDI), smoking status, alcohol consumption, and body mass index (BMI). *APOE*, apolipoprotein E; IR, incidence rate; HR, hazard ratio; CI, confidence interval; PRS, polygenic risk score.

There were no statistically significant interactions between neuroticism z‐score and *APOE* genotypes or non‐*APOE* PRS (*p* for interaction  > 0.05) (Figure 3). The associations between neuroticism z‐score and the risk of incident dementia and its subtypes remained consistent across all genetic subgroups.

### 3.3 Mediation analysis

**FIGURE 4:**
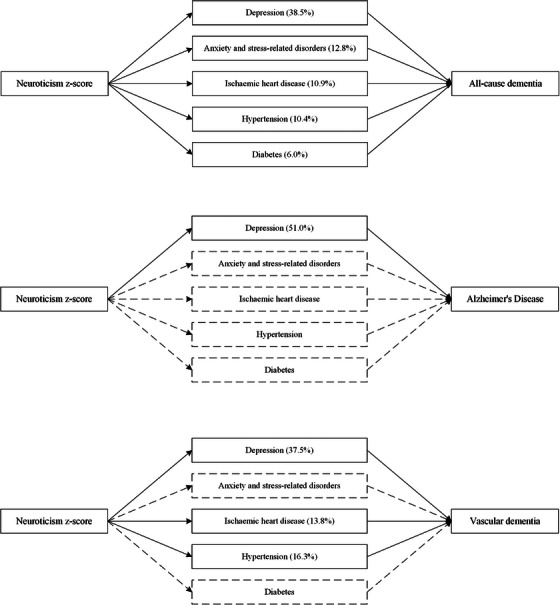
The association between neuroticism z‐score and dementia mediated by a history of diseases at baseline. Models were adjusted for age, sex, ethnicity, education, quintiles of the Townsend deprivation index (TDI), smoking status, alcohol consumption, and body mass index (BMI). The percentage values within brackets represent the proportion by which each mediator mediated the association between neuroticism z‐score and the dementia outcome. Mediators depicted with dashed borders and arrows indicate statistically insignificant mediation effects.

A history of depression, anxiety and stress‐related disorders, IHD, hypertension, and diabetes explained 38.5%, 12.8%, 10.9%, 10.4%, and 6.0% of the association between neuroticism z‐score and all‐cause dementia, respectively (Figure 4, eTable 8). Similar mediated proportions by depression were observed for VaD (37.5%), with IHD and hypertension mediating a larger proportion compared to the association with all‐cause dementia (10.9% and 10.4% vs 13.8% and 16.3%). Depression mediated a larger proportion of the association with AD than all‐cause dementia (51.0% vs 38.5%), whereas no mediation by IHD or hypertension was observed. There was no evidence of anxiety or diabetes mediating the association for both AD and VaD.

### 3.4 Association of neuroticism with neuroimaging outcomes and cognitive function

The characteristics of the 39,459 non‐demented individuals included in the neuroimaging and cognitive function analyses are presented in eTable 9. Neuroticism z‐score was found to be associated with lower total gray matter volume (β −0.01, 95% CI, −0.02 to 0.00; *p* = 0.038), and higher log‐WMH volume (β 0.02, 95% CI, 0.01 to 0.03; *p* = 1.6×10^−4^) (Table 2). No statistically significant association was observed between neuroticism z‐score and total brain volume, total white matter volume, and bilateral hippocampal volumes. Neuroticism z‐score was associated with worse function across all cognitive domains (all *p*’s < 0.001). In subgroup of participants ≥65 years of age (*n* = 20,945) and < 65 years (*n* = 18,514) at imaging assessment, the associations between neuroticism and most outcomes remained consistent with the main analysis, including WMH volume and cognitive function (eTable 10). However, the association with total gray matter volume, although in the same direction as the main analysis, was not statistically significant in either age group. Furthermore, neuroticism was positively associated with right hippocampal volume in those age <65 years, but not in those age ≥65.

**TABLE 2:** Association of neuroticism z‐score with neuroimaging outcomes and cognitive function.

	β (95% CI)	*p*
**Neuroimaging outcomes**		
Total brain volume	0.00 (−0.01 to 0.01)	0.806
Total white matter	0.01 (0.00 to 0.02)	0.160
Total gray matter	–0.01 (−0.02 to 0.00)	0.038
Total hippocampus	0.00 (−0.01 to 0.01)	0.643
Left hippocampus	0.00 (−0.01 to 0.01)	0.928
Right hippocampus	0.00 (−0.01 to 0.01)	0.468
White matter hyperintensities	0.02 (0.01 to 0.03)	1.6×10^−4^
**Cognitive function**		
Complex processing speed	–0.05 (−0.06 to −0.04)	<0.001
Verbal and numerical reasoning	–0.03 (−0.04 to −0.02)	<0.001
Non‐verbal reasoning	–0.03 (−0.04 to −0.02)	<0.001
Working memory	–0.03 (−0.05 to −0.02)	<0.001
Verbal declarative memory	–0.02 (−0.04 to −0.01)	<0.001
Executive function (Trail Making Test A)	–0.03 (−0.04 to −0.02)	<0.001
Executive function (Trail Making Test B)	–0.03 (−0.05 to −0.02)	<0.001

*Note*: Imaging‐related confounds (head size, head motion, head and table position, and imaging center) were regressed out from the neuroimaging outcomes (white matter hyperintensity [WMH] volumes were log‐transformed). Models were adjusted for age, sex, ethnicity, education, quintiles of the Townsend deprivation index (TDI), smoking status, alcohol consumption, body mass index (BMI). Sex, ethnicity, and TDI were measured at baseline, and for other covariates (age, education, smoking status, alcohol consumption, and BMI), measurements from the imaging study were used. CI, confidence interval.

## 4 DISCUSSION

In this large population‐based cohort, neuroticism was associated with an increased risk of all‐cause dementia regardless of genetic predisposition to dementia. The associations were largely explained by depression, anxiety, IHD, and hypertension. Higher neuroticism was associated with decreased total gray matter volume and increased WMH volume, and worse function across multiple cognitive domains.

Our results are consistent with previous meta‐analysis,^3^ which has found that one SD increase in neuroticism score (as measured by commonly used questionnaires such as EPQ and NEO Five‐Factor Inventory) was associated with a 24% greater risk of developing dementia (HR for one SD increase, 1.24 [95% CI, 1.17 to 1.31]). Terracciano et al. conducted a similar study using UKB based on 1798 incident all‐cause dementia cases with up to 12 years of follow‐up, and also found a positive association between neuroticism score and the risk of all‐cause dementia (HR for one SD increase, 1.18 [95% CI, 1.13 to 1.24]).^26^ Here, we extended the follow‐up to up to 15 years, and included ~4000 additional incident all‐cause dementia cases. Consequently, as the largest study investigating this association, we present robust evidence supporting the link between neuroticism and all‐cause dementia (HR for one SD increase, 1.11 [95% CI, 1.08 to 1.14]), as well as different dementia subtypes, by showing that the associations remain similar in more than 10 years of follow‐up. We also found evidence of a dose–response association, whereby each increase in neuroticism score corresponded to an increasing risk of dementia, with no evidence of any threshold effects.

Genetics are strongly implicated in dementia risk; thus it is important to understand whether this modifies the relationship between neuroticism and dementia risk. Only one previous study examined the interaction between neuroticism and genetic risks, identifying an interaction effect between *APOE* ε4 and neuroticism on AD (*N* = 86), but not on non‐AD dementia (*N* = 12), with up to 7 years of follow‐up.^27^ A longitudinal study (*N* = 912) found that *APOE* ε4 does not modify the association of neuroticism with cognitive ability and cognitive decline.^28^ In the current study, we found that neuroticism was consistently associated with dementia regardless of genetic predisposition to dementia based on either *APOE* ε4 carrier status or non‐*APOE* polygenic risk.

We found evidence that the associations were mediated by mental health conditions, specifically depression, as well as vascular conditions, including hypertension and IHD. Compared with other conditions, the proportion mediated via diabetes was smaller for all‐cause dementia, and there is no evidence that diabetes explains the association for AD and VaD, suggesting that metabolic conditions might not be the major contributors to the neuroticism–dementia association. This is expected, as diabetes was included as a “negative‐control.” Neuroticism has been linked with an increased risk of vascular conditions through behavioral choices, such as smoking,^29^ a poorer diet,^30^ and lower physical activity.^31^ Individuals with higher levels of neuroticism often exhibit reduced ability to cope with stress, which in turn is linked to poorer vascular health. A previous study exploring the association between neuroticism and dementia found that the associations substantially attenuated when long‐standing stressor‐related distress (measured by feelings of irritability, anxiety, or sleep disturbances lasting for 1 month or longer due to stress) is included in the model.^32^ Stress exposure disrupts activity of the hypothalamic–pituitary–adrenal (HPA) axis and increases inflammation, which is particularly detrimental to the vascular system.^33^ Supporting this, higher neuroticism scores have been associated with elevated cortisol levels in older adults.^34^ Behavioral interventions for reducing neuroticism show promise but are often limited by small‐scale studies,^35^ selective samples (e.g., university students),^36^, ^37^ and lengthy sessions,^38^ impacting their robustness and scalability. Our findings suggest that targeting mental and vascular health conditions could be an alternative approach for dementia risk reduction within individuals with higher neuroticism.

We observed that the association between neuroticism and VaD was stronger than that with AD. However, distinguishing between different dementia subtypes can be challenging as we rely on ICD codes recorded in administrative data.^39^ However, to complement these results, we also found that neuroticism was associated with increased cerebrovascular burden in a dementia‐free neuroimaging subset. This aligns with a prior study showing a positive association between neuroticism and WMH volume.^40^ We also observed a significant association between neuroticism and total gray matter atrophy, but not hippocampal atrophy, which is a hallmark of AD. Several studies, consisting of 200–600 participants of older age, similarly failed to identify a significant association between neuroticism and hippocampal volume.^41^, ^42^ Taken together, these findings suggest that neuroticism may be particularly strongly associated with cerebrovascular pathology, rather than acting through a widespread mechanism that affects structures of dementia‐related brain regions. We also found statistically significant associations between neuroticism and worse cognitive function across all, rather than specific, domains. This extends previous findings that identified the association between neuroticism and worse global cognitive outcomes,^43^ and enhances the reliability of previous studies (*N* = 179,^44^ *N* = 2865^45^) that demonstrated the association between neuroticism and worse cognitive function in multiple domains (memory, executive function, language, and visual‐spatial function).

Strengths of this study include the large sample size, number of dementia cases, long follow‐up, and novel analyses investigating the role of genetic predisposition to dementia, mediating factors, and associations with neuroimaging outcomes and cognitive function. However, our study has several limitations to consider. We identified dementia cases through hospital records and death registry data, likely missing less severe cases diagnosed in other settings. This misclassification of outcomes may have biased associations toward the null. UKB achieved only a 5.5% response rate, recruiting 0.5 million participants for baseline assessment out of 9 million invited.^46^ UKB participants,^47^ especially those in the imaging study,^48^ are generally healthier and of higher socioeconomic status compared to the wider UK population. The non‐*APOE* PRS we developed was based on GWAS on AD, rather than all‐cause dementia or VaD. Exposure and mediator measurements lack temporal separation. As with all observational studies, causality cannot be inferred and residual confounding likely remains. Further studies that validate these findings in other large and diverse cohorts with extended follow‐up and mediators measured after the exposure and prior to the outcome are warranted.

In conclusion, our study suggests that high levels of neuroticism are associated with an elevated risk of dementia, in particular vascular dementia (or VaD), accompanied by an increased burden of brain vascular pathology and worse cognitive function. It is notable that this association remains significant regardless of genetic risk. Despite neuroticism being a stable personality trait, which is not easily modifiable, evaluating neuroticism may serve as a means to identify individuals at high risk for dementia. Furthermore, our findings suggest that the associations between neuroticism and dementia are driven largely by mental and vascular conditions, implying that addressing the increased burden of mental and vascular health conditions in individuals with higher neuroticism could, if causal, ultimately help prevent or delay the onset of dementia.

## CONFLICT OF INTEREST STATEMENT

The authors have nothing to disclose. Author disclosures are available in the supporting information.

## CONSENT STATEMENT

All human subjects provided informed consent via electronic signature through the touchscreen.

## Supporting information

Supporting information

Supporting information
