# Supplementary material for: Association of neuroticism with incident dementia, neuroimaging outcomes, and cognitive function
Source: Alzheimers Dement. 2024 Jul 10;20(8):5578–89. doi: 10.1002/alz.14071 (PMC11350007; doi:10.1002/alz.14071)
Supplement: Supplementary file 1 — Supporting information [file ALZ-20-5578-s002.docx]

**Supplement**

Contents

[eTable 1. Baseline characteristics of participants with and without missing neuroticism data	0](#_Toc164065565)

[eTable 2. Questions and corresponding neurotic traits in the Eysenck Personality Questionnaire-Revised Short Form (EPQ-RS)	1](#_Toc164065566)

[eTable 3. ICD-9 and ICD-10 codes in the hospital inpatient records and death registries	2](#_Toc164065567)

[eFigure 1. Distribution of neuroimaging outcomes	3](#_Toc164065568)

[eTable 4. Definition of variables included in the UK Biobank data showcase	4](#_Toc164065569)

[eMethods. Multiple imputation	7](#_Toc164065570)

[eMethods. Genetic risk assessment	8](#_Toc164065571)

[eMethods. Mediation analyses	9](#_Toc164065572)

[eTable 5. Baseline characteristics of participants by dementia diagnoses	10](#_Toc164065573)

[eFigure 2. Associations between neuroticism score and incident subtypes of dementia	12](#_Toc164065574)

[eFigure 3. Association between neuroticism z-score and incident dementia by different follow-up periods	13](#_Toc164065575)

[eTable 6. Association between neuroticism z-score and incident dementia after multiple imputation	14](#_Toc164065576)

[eTable 7. Association between neuroticism z-score and incident dementia in all participants without dementia, regardless of baseline age	16](#_Toc164065577)

[eTable 8. Estimated direct and indirect effect sizes of neuroticism z-score with dementia through prevalent diseases at baseline	18](#_Toc164065578)

[eTable 9. Main characteristics of the participants included in the neuroimaging and cognitive function analyses	19](#_Toc164065579)

[eTable 10. Association of neuroticism z-score with neuroimaging outcomes and cognitive function stratified by age at the imaging assessment	20](#_Toc164065580)

## eTable 1. Baseline characteristics of participants with and without missing neuroticism data

| **Characteristic** | **Non-missing** | **Missing** |
| --- | --- | --- |
| **N** | 174164 | 45321 |
| **Age (mean (SD))** | 64.36 (2.96) | 64.66 (3.01) |
| **Male (%)** | 83849 (48.1) | 19313 (42.6) |
| **Townsend deprivation index quintile** |  |  |
| 1 (Least deprived) | 38198 (21.9) | 9221 (20.3) |
| 2 | 38197 (21.9) | 9272 (20.5) |
| 3 | 36256 (20.8) | 9291 (20.5) |
| 4 | 32841 (18.9) | 8789 (19.4) |
| 5 (Most deprived) | 28672 (16.5) | 8748 (19.3) |
| **Education (%)** |  |  |
| Primary | 44741 (25.7) | 13875 (30.6) |
| Secondary | 36128 (20.7) | 9198 (20.3) |
| Post-secondary non-tertiary | 24329 (14.0) | 5901 (13.0) |
| Tertiary | 68966 (39.6) | 16347 (36.1) |
| **Ethnic group - White (%)** | 170011 (97.6) | 43236 (95.4) |
| **BMI (%)** |  |  |
| Normal | 51858 (29.8) | 13992 (30.9) |
| Overweight | 78883 (45.3) | 20063 (44.3) |
| Obese | 43423 (24.9) | 11266 (24.9) |
| **Drinking daily or almost daily (%)** | 132224 (24.1) | 9629 (21.2) |
| **Smoking status (%)** |  |  |
| Never | 86655 (49.8) | 23404 (51.6) |
| Previous | 73118 (42.0) | 18185 (40.1) |
| Current | 14391 ( 8.3) | 3732 ( 8.2) |
| ***APOE* genotypes (%)** |  |  |
| *APOE* ε2 carriers | 22236 (12.8) | 5676 (12.5) |
| *APOE* ε3/ ε3 carriers | 100368 (57.6) | 26079 (57.5) |
| *APOE* ε4 carriers | 43683 (25.1) | 11453 (25.3) |
| Missing/Ambiguous genotypes^a^ | 7877 ( 4.5) | 2113 ( 4.7) |
| **Non-*APOE* PRS^b^ (%)** |  |  |
| Low | 27996 (16.1) | 7321 (16.2) |
| Intermediate | 84795 (48.7) | 22114 (48.8) |
| High | 28559 (16.4) | 7373 (16.3) |
| Missing | 32814 (18.8) | 8513 (18.8) |
| **Medical history (%)** |  |  |
| Depression | 8744 ( 5.0) | 2594 ( 5.7) |
| Anxiety and stress disorder | 2800 ( 1.6) | 790 ( 1.7) |
| Hypertension | 64338 (36.9) | 17618 (38.9) |
| Cardiovascular diseases | 15176 ( 8.7) | 4122 ( 9.1) |
| Diabetes | 12083 ( 6.9) | 3330 ( 7.3) |

N, number of participants; SD, Standard deviation; BMI, body mass index.

^a^ *APOE* ε1/ε3 and *APOE* ε2/ε4 genotypes.

^b^ Low (lowest PRS quintile), intermediate (PRS quintiles 2 to 4), and high (highest PRS quintile).

## eTable 2. Questions and corresponding neurotic traits in the Eysenck Personality Questionnaire-Revised Short Form (EPQ-RS)

| **Neurotic trait** | **Item** | **UK Biobank field ID** |
| --- | --- | --- |
| Mood swings | Does your mood often go up and down? | 1920 |
| Miserableness | Do you ever feel 'just miserable' for no reason? | 1930 |
| Irritability | Are you an irritable person? | 1940 |
| Sensitivity | Are your feelings easily hurt? | 1950 |
| Fed-up feelings | Do you often feel 'fed-up'? | 1960 |
| Nervous feelings | Would you call yourself a nervous person? | 1970 |
| Worry | Are you a worrier? | 1980 |
| Tense | Would you call yourself tense or 'highly strung'? | 1990 |
| Persistent worry after embarrassment | Do you worry too long after an embarrassing experience? | 2000 |
| Suffer from nerves | Do you suffer from 'nerves'? | 2010 |
| Loneliness | Do you often feel lonely? | 2020 |
| Guilty feelings | Are you often troubled by feelings of guilt? | 2030 |

## eTable 3. ICD-9 and ICD-10 codes in the hospital inpatient records and death registries

| **Disease** | **ICD-9 codes** | **ICD-10 codes** |
| --- | --- | --- |
| All-cause dementia | 2902, 2903, 2904, 2912, 2941, 3310, 3311, 3312, 3315 | A810, F00, F01, F02, F03, F051, F106, G30, G310, G311, G318, I673 |
| Alzheimer’s disease | 3310 | F00, G30 |
| Vascular dementia | 2904 | F01, I673 |
| Depression | 2962, 2963, 29682 | F32, F33 |
| Anxiety and stress-related disorders | 3000, 3002, 308, 309 | F40, F41, F43 |
| Diabetes | 250 | E10, E11, E13 |
| Hypertension | 401-405 | I10-I15 |
| Ischaemic heart disease | 410-414 | I20-I25 |

ICD, International Classification of Diseases

## eFigure 1. Distribution of neuroimaging outcomes

The visualizations of these distributions were created using the 'geom_density' function from the 'ggplot2' package in R. The 'geom_density' function makes use of the histogram of the original data to compute a kernel density estimate, which is then plotted.

## eTable 4. Definition of variables included in the UK Biobank data showcase

| **Variable** | **UK Biobank field ID(s)** | **Measurement item(s)** | **Definition/categorization** |
| --- | --- | --- | --- |
| Age | 34, 53 | (Registry) year of birth and assessment | Age = year of assessment – year of birth |
| Sex | 31 | Registry |  |
| Ethnicity | 21000 | (Questionnaire) amalgam of sequential branching questions | We used the first non-missing value across all measurement occasions.   1) White; 2) Non-white (Asian or Asian British, Black or Black British, Chinese, Mixed, and other ethnic group) |
| Education | 6138 | (Questionnaire) self-reported qualifications | Participants with multiple qualifications were assigned the highest qualification attained, which was subsequently converted to the International Standard Classification for Education (ISCED) code representing years of education:^1,2^   1) Tertiary: College/University Degree, National Vocational Qualification (NVQ) / Higher National Diploma (HND) / Higher National Certificate (HNC) or equivalent 2) Post-secondary non-tertiary: Other professional qualifications (e.g., nursing, teaching) 3) Secondary: General Certificate of Education Advanced Level (A levels) / General Certificate of Education Advanced Subsidiary Level (AS levels) or equivalent, General Certificate of Education Ordinary Level (O levels) / General Certificate of Secondary Education (GCSEs) or equivalent, Certificate of Secondary Education (CSEs) or equivalent 4) Primary: None of the above |
| Townsend deprivation index (TDI) | 189 | The TDI was computed for each participant using the national census data preceding their enrolment in UK Biobank. The score was assigned based on the output area of the participant's postcode. | A greater Townsend index score implies a greater degree of deprivation. The entire UK Biobank sample was categorized into quintiles based on their TDI, with quintile 1 representing the richest and quintile 5 representing the poorest. |
| Smoking status | 20116 | (Questionnaire) self-reported smoking-status | 1) Never; 2) Previous; 3) Current |
| Alcohol consumption | 1558 | (Questionnaire) self-reported drinking frequency | 1) ≤ 4 times week; 2) Daily or almost daily ^3^ |
| Body mass index (BMI) | 21001 | (Physical measures) height and weight | The classification of BMI is utilized by the World Health Organization:^4^   1) Normal (<25 kg/m^2^); 2) Overweight (25–30 kg/m^2^); 3) Obese (≥30 kg/m^2^) |
| History of dementia | 20002 | (Verbal Interview) self-reported illness | "dementia/alzheimers/cognitive impairment" |
| History of depression | 20002 | (Verbal Interview) self-reported illness | "depression" |
| History of anxiety and stress-related disorders | 20002 | (Verbal Interview) self-reported illness | "anxiety/panic attacks", "post-traumatic stress disorder" |
| History of diabetes | 20002 | (Verbal Interview) self-reported illness | "diabetes", "type 1 diabetes", "type 2 diabetes" |
| History of hypertension | 20002 | (Verbal Interview) self-reported illness | "hypertension", "essential hypertension" |
| History of ischaemic heart disease | 20002 | (Verbal Interview) self-reported illness | "angina", "heart attack/myocardial infarction" |
| Imaging site | 54 | Registry |  |
| Head size | 25000 | T1 structural brain MRI | Volumetric scaling from T1 head image to standard space |
| Head position | 25756, 25757, 25758 | T1 structural brain MRI | X, Y, Z positions of the centre-of-gravity of the brain mask in scanner coordinates |
| Table position | 25759 | T1 structural brain MRI | Table position in scanner coordinates |
| Head motion | 24419 | T1 structural brain MRI |  |
| Volume of total brain | 25010 | T1 structural brain MRI |  |
| Volume of white matter | 25008 | T1 structural brain MRI |  |
| Volume of grey matter | 25006 | T1 structural brain MRI |  |
| Volume of hippocampus | 25019, 25020 | T1 structural brain MRI |  |
| Volume of white matter hyperintensities | 25781 | T1 and T2 FLAIR images |  |
| Fluid intelligence | 20016 | Cognitive function | Number of questions answered correctly in two minutes |
| Backward digit span task | 4282 | Cognitive function | The maximum number of digits correctly remembered |
| Trail making test A | 6348 | Cognitive function | Duration to complete numeric path |
| Trail making test B | 6350 | Cognitive function | Duration to complete alphanumeric path |
| Symbol digit substitution | 23324 | Cognitive function | Number of correct symbol-digit matches made in 60 seconds |
| Paired associate learning | 20197 | Cognitive function | Number of word pairs correctly associated |
| Matrix pattern completion | 6373 | Cognitive function | Number of puzzles correctly solved in 3 minutes |

## eMethods. Multiple imputation

We used multiple imputation by chained equations to impute missing neuroticism score and covariates. The imputation model included the 12 Eysenck Personality Questionnaire-Revised Short Form (EPQ-RS) items, all preselected covariates, the Nelson-Aalen estimate of cumulative baseline hazard, and dementia status.^5^ The imputed item scores were then added and standardized to form a z-score.^6^ We conducted Cox proportional-hazards regression analyses to assess the association between both neuroticism original score and z-score with incident dementia and its subtypes in five imputed datasets, and the results from each of the five imputed datasets were combined using Rubin’s rules.^7^

## eMethods. Genetic risk assessment

*APOE* genotypes were estimated from 2 single nucleotide polymorphisms (SNP), rs7412 and rs429358. Participants with at least one copy of *APOE* ε2 were considered *APOE* ε2 carriers, those with at least one copy of *APOE* ε4 were considered *APOE* ε4 carriers, and *APOE* ε3/ε3 was used as the reference group. Ambiguous genotypes, *APOE* ε1/ ε3 and *APOE* ε2/ε4, were excluded.

We calculated dementia non-*APOE* polygenic risk score (PRS) based on a 39-SNP PRS developed by Ebenau et al,^8^ which predicted AD dementia independently of *APOE* ε4. Ebenau et al selected those SNPs from genome‐wide association studies (GWAS) for late-onset AD, and derived variant-specific weight from International Genomics of Alzheimer's Project studies. For this analysis, we performed quality controls by excluding one SNP that had minor allele frequency <0.005, leaving 38 SNPs for PRS calculation. The PRS was then divided into quintiles, and participants were categorized into three genetic risk groups: low (lowest PRS quintile), intermediate (PRS quintiles 2 to 4), and high (highest PRS quintile).

## eMethods. Mediation analyses

In this study, we employed the mediation analysis approach proposed by Lange et al.^9^ to estimate the extent to which the association between neuroticism z-score (exposure, denoted as "A") and incident dementia risk (outcome, denoted as "Y") was mediated by the history of the following diseases (potential mediators, denoted as "M"): depression, anxiety and stress-related disorders, cardiovascular disease, hypertension, and diabetes (excluding gestational diabetes). Self-reported medical conditions during the verbal interview and hospital diagnoses prior to baseline were used to ascertain the baseline disease histories. Table S2 – S3 provide the relevant codes for hospital diagnoses (International Classification of Diseases codes) and self-report used to identify these histories. We also adjusted for preselected covariates, including age, sex, ethnicity, education, Townsend deprivation index (TDI) quintiles, smoking status, alcohol consumption, and body mass index (covariates, denoted as "C").

This approach decomposes the total effect into natural direct effects and natural indirect effects within a counterfactual framework. Let Y_a,m_ denote the outcome value when A and M are set to A = a and M = m, respectively. The natural direct effect is defined as Y_a,M(a*)_ – Y_a*,M(a*)_, where a ≠ a*, i.e., the difference between the outcome value under exposure A = a and the outcome value if the same individual were instead exposed to A = a* with the mediator fixed at the level it would take under exposure level a*. The natural indirect effect is defined as Y_a,M(a)_ – Y_a,M(a*)_, reflecting the change in the outcome value when the exposure is fixed at level A = a, and the mediator is changed to its level under exposure A = a*. At the population level, the natural direct effect describes the effect of A on Y that is not due to changes in M, where M can vary among individuals according to all determinants except for A. Conversely, the natural indirect effect describes the effect of A on Y that is transmitted through M.

Marginal structural models (MSMs) are used to model the marginal expectation of a counterfactual outcome, which is implemented through the following steps:

(1) Fitting logistic regression models to estimate the association between each potential mediator (history of a specific disease) and neuroticism z-score, conditioned on baseline covariates.
(2) Constructing a new dataset by repeating each observation in the original data 5 times and replacing A* for subject *i* by randomly drawn exposures through resampling from the observed exposures.
(3) Calculating the weight for each observation in the new dataset using the fitted models from step 1.

$$W_{i}^{C}=\frac{P\left( M=M_{i} | A=A_{i}^{*}, C=C_{i} \right)}{P\left( M=M_{i} | A=A_{i}, C=C_{i} \right)}$$

(4) Fitting a weighted Cox model to estimate the hazard ratios (HR) for the direct and indirect effects, while controlling for baseline covariates. To account for the correlation between repeated observations, robust standard errors were calculated.
(5) Calculating the proportion of the total effect that is explained by each individual mediator as HR_indirect effect_ / HR_total effect_.

## eTable 5. Baseline characteristics of participants by dementia diagnoses

| **Characteristic** | **No incident dementia** | **Incident dementia** | | |
| --- | --- | --- | --- | --- |
|  |  | **all-cause dementia** | **Alzheimer's Disease** | **Vascular Dementia** |
| **N** | 168190 | 5974 | 2741 | 1364 |
| **Age (mean (SD))** | 64.30 (2.95) | 66.01 (2.82) | 66.13 (2.80) | 66.08 (2.80) |
| **Male (%)** | 80702 (48.0) | 3147 (52.7) | 1313 (47.9) | 812 (59.5) |
| **Townsend deprivation index quintile** |  |  |  |  |
| 1 (Least deprived) | 37017 (22.0) | 1181 (19.8) | 579 (21.1) | 259 (19.0) |
| 2 | 37005 (22.0) | 1192 (20.0) | 546 (19.9) | 260 (19.1) |
| 3 | 35121 (20.9) | 1135 (19.0) | 504 (18.4) | 256 (18.8) |
| 4 | 31685 (18.8) | 1156 (19.4) | 508 (18.5) | 275 (20.2) |
| 5 (Most deprived) | 27362 (16.3) | 1310 (21.9) | 604 (22.0) | 314 (23.0) |
| **Education (%)** |  |  |  |  |
| Primary | 42586 (25.3) | 2155 (36.1) | 1014 (37.0) | 521 (38.2) |
| Secondary | 34973 (20.8) | 1155 (19.3) | 522 (19.0) | 258 (18.9) |
| Post-secondary non-tertiary | 23607 (14.0) | 722 (12.1) | 329 (12.0) | 164 (12.0) |
| Tertiary | 67024 (39.9) | 1942 (32.5) | 876 (32.0) | 421 (30.9) |
| **Ethnic group - White (%)** | 164207 (97.6) | 5804 (97.2) | 2658 (97.0) | 1328 (97.4) |
| **BMI (%)** |  |  |  |  |
| Normal | 50072 (29.8) | 1786 (29.9) | 874 (31.9) | 332 (24.3) |
| Overweight | 76288 (45.4) | 2595 (43.4) | 1208 (44.1) | 572 (41.9) |
| Obese | 41830 (24.9) | 1593 (26.7) | 659 (24.0) | 460 (33.7) |
| **Drinking daily or almost daily (%)** | 40691 (24.2) | 1249 (20.9) | 547 (20.0) | 277 (20.3) |
| **Smoking status (%)** |  |  |  |  |
| Never | 83932 (49.9) | 2723 (45.6) | 1311 (47.8) | 563 (41.3) |
| Previous | 70420 (41.9) | 2698 (45.2) | 1201 (43.8) | 645 (47.3) |
| Current | 13838 ( 8.2) | 553 ( 9.3) | 229 ( 8.4) | 156 (11.4) |
| ***APOE* genotypes (%)** |  |  |  |  |
| *APOE* ε2 carriers | 21816 (13.0) | 420 ( 7.0) | 142 ( 5.2) | 114 ( 8.4) |
| *APOE* ε3/ ε3 carriers | 98085 (58.3) | 2283 (38.2) | 867 (31.6) | 555 (40.7) |
| *APOE* ε4 carriers | 40718 (24.2) | 2965 (49.6) | 1588 (57.9) | 623 (45.7) |
| Missing/Ambiguous genotypes^a^ | 7571 ( 4.5) | 306 ( 5.1) | 144 ( 5.3) | 72 ( 5.3) |
| **Non-*APOE* PRS^b^ (%)** |  |  |  |  |
| Low | 27264 (16.2) | 732 (12.3) | 281 (10.3) | 189 (13.9) |
| Intermediate | 81983 (48.7) | 2812 (47.1) | 1269 (46.3) | 634 (46.5) |
| High | 27286 (16.2) | 1273 (21.3) | 655 (23.9) | 275 (20.2) |
| Missing | 31657 (18.8) | 1157 (19.4) | 536 (19.6) | 266 (19.5) |
| **Medical history (%)** |  |  |  |  |
| Depression | 8248 ( 4.9) | 496 ( 8.3) | 216 ( 7.9) | 123 ( 9.0) |
| Anxiety and stress disorder | 2643 ( 1.6) | 157 ( 2.6) | 70 ( 2.6) | 37 ( 2.7) |
| Hypertension | 61527 (36.6) | 2811 (47.1) | 1201 (43.8) | 792 (58.1) |
| Cardiovascular diseases | 14227 ( 8.5) | 949 (15.9) | 364 (13.3) | 289 (21.2) |
| Diabetes | 11250 ( 6.7) | 833 (13.9) | 334 (12.2) | 293 (21.5) |
| **Neuroticism z-score (mean (SD))** | 0.00 (1.00) | 0.08 (1.04) | 0.05 (1.03) | 0.10 (1.04) |

N, number of participants; SD, Standard deviation; BMI, body mass index; PRS, polygenic risk score.

^a^ *APOE* ε1/ε3 and *APOE* ε2/ε4 genotypes.

^b^ Low (lowest PRS quintile), intermediate (PRS quintiles 2 to 4), and high (highest PRS quintile).

## eFigure 2. Associations between neuroticism score and incident subtypes of dementia

(A) Association between neuroticism score and incident Alzheimer’s disease. (B) Association between neuroticism score and vascular dementia. Models were adjusted for age, sex, ethnicity, education, quintiles of the Townsend deprivation index (TDI), smoking status, alcohol consumption, and body mass index (BMI). HR, hazard ratio. CI, confidence interval.

## eFigure 3. Association between neuroticism z-score and incident dementia by different follow-up periods

Models were adjusted for age, sex, ethnicity, education, quintiles of the Townsend deprivation index (TDI), smoking status, alcohol consumption, and body mass index (BMI). IR, incidence rate. HR, hazard ratio. CI, confidence interval.

## eTable 6. Association between neuroticism z-score and incident dementia after multiple imputation

| **Neuroticism score** | **HR (95% CI)** | ***p*** |
| --- | --- | --- |
| **All-cause dementia** |  |  |
| 0 | 1 (reference) |  |
| 1 | 1.05 (0.96 to 1.15) | 0.281 |
| 2 | 1.14 (1.04 to 1.24) | 0.007 |
| 3 | 1.04 (0.94 to 1.15) | 0.428 |
| 4 | 1.10 (0.99 to 1.21) | 0.067 |
| 5 | 1.14 (1.03 to 1.26) | 0.013 |
| 6 | 1.22 (1.10 to 1.36) | <0.001 |
| 7 | 1.21 (1.08 to 1.35) | <0.001 |
| 8 | 1.33 (1.18 to 1.50) | <0.001 |
| 9 | 1.21 (1.05 to 1.39) | 0.008 |
| 10 | 1.56 (1.36 to 1.79) | <0.001 |
| 11 | 1.59 (1.36 to 1.85) | <0.001 |
| 12 | 1.75 (1.48 to 2.08) | <0.001 |
| Neuroticism z-score | 1.12 (1.10 to 1.15) | <0.001 |
| **Alzheimer’s disease** |  |  |
| 0 | 1 (reference) |  |
| 1 | 0.96 (0.84 to 1.10) | 0.563 |
| 2 | 1.01 (0.88 to 1.15) | 0.934 |
| 3 | 0.97 (0.84 to 1.12) | 0.666 |
| 4 | 1.02 (0.88 to 1.18) | 0.785 |
| 5 | 1.00 (0.86 to 1.16) | 0.997 |
| 6 | 1.10 (0.94 to 1.29) | 0.224 |
| 7 | 1.07 (0.91 to 1.27) | 0.422 |
| 8 | 1.09 (0.90 to 1.31) | 0.377 |
| 9 | 1.15 (0.94 to 1.41) | 0.179 |
| 10 | 1.34 (1.09 to 1.66) | 0.006 |
| 11 | 1.21 (0.94 to 1.56) | 0.13 |
| 12 | 1.31 (0.99 to 1.73) | 0.056 |
| Neuroticism z-score | 1.07 (1.03 to 1.11) | <0.001 |
| **Vascular dementia** |  |  |
| 0 | 1 (reference) |  |
| 1 | 1.21 (0.99 to 1.48) | 0.056 |
| 2 | 1.23 (1.01 to 1.50) | 0.042 |
| 3 | 1.13 (0.91 to 1.39) | 0.265 |
| 4 | 1.20 (0.97 to 1.49) | 0.098 |
| 5 | 1.32 (1.06 to 1.64) | 0.012 |
| 6 | 1.34 (1.07 to 1.68) | 0.012 |
| 7 | 1.26 (0.98 to 1.61) | 0.072 |
| 8 | 1.86 (1.46 to 2.36) | <0.001 |
| 9 | 1.42 (1.06 to 1.91) | 0.02 |
| 10 | 1.52 (1.11 to 2.09) | 0.01 |
| 11 | 1.65 (1.16 to 2.33) | 0.005 |
| 12 | 2.15 (1.52 to 3.06) | <0.001 |
| Neuroticism z-score | 1.16 (1.10 to 1.22) | <0.001 |

Models were adjusted for age, sex, ethnicity, education, quintiles of the Townsend deprivation index (TDI), smoking status, alcohol consumption, and body mass index (BMI). HR, hazard ratio. CI, confidence interval.

## eTable 7. Association between neuroticism z-score and incident dementia in all participants without dementia, regardless of baseline age

| **Neuroticism score** | **HR (95% CI)** | ***p*** |
| --- | --- | --- |
| **All-cause dementia** |  |  |
| 0 | 1 (reference) |  |
| 1 | 1.07 (0.98 to 1.17) | 0.136 |
| 2 | 1.13 (1.04 to 1.23) | 0.004 |
| 3 | 1.07 (0.97 to 1.18) | 0.165 |
| 4 | 1.14 (1.04 to 1.25) | 0.006 |
| 5 | 1.15 (1.03 to 1.28) | 0.013 |
| 6 | 1.22 (1.09 to 1.36) | <0.001 |
| 7 | 1.17 (1.05 to 1.31) | 0.005 |
| 8 | 1.27 (1.13 to 1.42) | <0.001 |
| 9 | 1.26 (1.10 to 1.44) | <0.001 |
| 10 | 1.51 (1.31 to 1.75) | <0.001 |
| 11 | 1.48 (1.24 to 1.78) | <0.001 |
| 12 | 1.64 (1.37 to 1.97) | <0.001 |
| Neuroticism z-score | 1.10 (1.08 to 1.13) | <0.001 |
| **Alzheimer’s disease** |  |  |
| 0 | 1 (reference) |  |
| 1 | 1.02 (0.89 to 1.17) | 0.779 |
| 2 | 1.04 (0.92 to 1.18) | 0.536 |
| 3 | 0.99 (0.86 to 1.14) | 0.878 |
| 4 | 1.07 (0.94 to 1.23) | 0.295 |
| 5 | 1.06 (0.92 to 1.22) | 0.395 |
| 6 | 1.07 (0.92 to 1.24) | 0.357 |
| 7 | 1.05 (0.87 to 1.26) | 0.594 |
| 8 | 1.05 (0.87 to 1.27) | 0.624 |
| 9 | 1.14 (0.94 to 1.37) | 0.177 |
| 10 | 1.23 (0.96 to 1.56) | 0.098 |
| 11 | 1.12 (0.86 to 1.46) | 0.398 |
| 12 | 1.42 (1.10 to 1.83) | 0.007 |
| Neuroticism z-score | 1.05 (1.01 to 1.08) | 0.005 |
| **Vascular dementia** |  |  |
| 0 | 1 (reference) |  |
| 1 | 1.26 (1.05 to 1.52) | 0.013 |
| 2 | 1.27 (1.05 to 1.55) | 0.015 |
| 3 | 1.22 (1.00 to 1.49) | 0.045 |
| 4 | 1.23 (1.00 to 1.53) | 0.053 |
| 5 | 1.39 (1.13 to 1.70) | 0.002 |
| 6 | 1.36 (1.08 to 1.71) | 0.009 |
| 7 | 1.32 (1.06 to 1.65) | 0.013 |
| 8 | 1.79 (1.43 to 2.24) | <0.001 |
| 9 | 1.60 (1.21 to 2.12) | 0.001 |
| 10 | 1.69 (1.25 to 2.29) | <0.001 |
| 11 | 1.95 (1.43 to 2.64) | <0.001 |
| 12 | 2.09 (1.46 to 2.98) | <0.001 |
| Neuroticism z-score | 1.16 (1.11 to 1.22) | <0.001 |

Models were adjusted for age, sex, ethnicity, education, quintiles of the Townsend deprivation index (TDI), smoking status, alcohol consumption, and body mass index (BMI). HR, hazard ratio. CI, confidence interval.

## eTable 8. Estimated direct and indirect effect sizes of neuroticism z-score with dementia through prevalent diseases at baseline

| **Mediator** | **Total effect** | **Direct effect** | **Indirect effect** | **% Mediated** |
| --- | --- | --- | --- | --- |
| **All-cause dementia** |  |  |  |  |
| Depression | 1.11 (1.08 to 1.14) | 1.07 (1.04 to 1.10) | 1.04 (1.02 to 1.06) | 38.5% |
| Anxiety and stress-related disorders | 1.11 (1.08 to 1.14) | 1.10 (1.07 to 1.13) | 1.01 (1.00 to 1.03) | 12.8% |
| Ischaemic heart disease | 1.11 (1.08 to 1.14) | 1.10 (1.07 to 1.13) | 1.01 (1.00 to 1.02) | 10.9% |
| Hypertension | 1.11 (1.08 to 1.14) | 1.10 (1.07 to 1.13) | 1.01 (1.00 to 1.02) | 10.4% |
| Diabetes | 1.11 (1.08 to 1.14) | 1.10 (1.08 to 1.13) | 1.01 (1.00 to 1.02) | 6.0% |
| **Alzheimer’s disease** |  |  |  |  |
| Depression | 1.05 (1.01 to 1.10) | 1.03 (0.98 to 1.07) | 1.03 (1.01 to 1.05) | 51.0% |
| Anxiety and stress-related disorders | 1.06 (1.01 to 1.10) | 1.04 (1.00 to 1.09) | 1.01 (0.99 to 1.03) | NA |
| Ischaemic heart disease | 1.05 (1.01 to 1.10) | 1.05 (1.01 to 1.09) | 1.00 (0.99 to 1.02) | NA |
| Hypertension | 1.05 (1.01 to 1.10) | 1.05 (1.01 to 1.09) | 1.00 (0.99 to 1.02) | NA |
| Diabetes | 1.05 (1.01 to 1.10) | 1.06 (1.02 to 1.10) | 1.00 (0.98 to 1.01) | NA |
| **Vascular dementia** |  |  |  |  |
| Depression | 1.15 (1.09 to 1.22) | 1.09 (1.03 to 1.16) | 1.06 (1.02 to 1.09) | 37.5% |
| Anxiety and stress-related disorders | 1.16 (1.10 to 1.23) | 1.14 (1.08 to 1.20) | 1.02 (0.99 to 1.04) | NA |
| Ischaemic heart disease | 1.15 (1.09 to 1.22) | 1.13 (1.07 to 1.19) | 1.02 (1.00 to 1.04) | 13.8% |
| Hypertension | 1.15 (1.09 to 1.22) | 1.12 (1.07 to 1.18) | 1.02 (1.00 to 1.05) | 16.3% |
| Diabetes | 1.15 (1.08 to 1.21) | 1.13 (1.08 to 1.20) | 1.01 (0.99 to 1.03) | NA |

Models were adjusted for age, sex, ethnicity, education, quintiles of the Townsend deprivation index (TDI), smoking status, alcohol consumption, and body mass index (BMI). In cases where the indirect effect was statistically insignificant, the corresponding proportion mediated was not calculated and is indicated as 'NA' (not applicable) in the table.

## eTable 9. Main characteristics of the participants included in the neuroimaging and cognitive function analyses

| **Characteristic** | **Overall** | **Neuroticism score tertile** | | |
| --- | --- | --- | --- | --- |
|  |  | **1** | **2** | **3** |
| **N** | 39459 | 13153 | 13153 | 13153 |
| **Age ^a^ (mean (SD))** | 64.79 (7.75) | 65.78 (7.61) | 65.11 (7.68) | 63.46 (7.76) |
| **Male (%)** | 19761 (50.1) | 7917 (60.2) | 6384 (48.5) | 5460 (41.5) |
| **Townsend deprivation index quintile** |  |  |  |  |
| 1 (Least deprived) | 9551 (24.2) | 3314 (25.2) | 3222 (24.5) | 3015 (22.9) |
| 2 | 9061 (23.0) | 3018 (22.9) | 3101 (23.6) | 2942 (22.4) |
| 3 | 8219 (20.8) | 2767 (21.0) | 2730 (20.8) | 2722 (20.7) |
| 4 | 7331 (18.6) | 2406 (18.3) | 2380 (18.1) | 2545 (19.3) |
| 5 (Most deprived) | 5297 (13.4) | 1648 (12.5) | 1720 (13.1) | 1929 (14.7) |
| **Education^a^ (%)** |  |  |  |  |
| Primary | 2486 ( 6.3) | 799 ( 6.1) | 793 ( 6.0) | 894 ( 6.8) |
| Secondary | 7402 (18.8) | 2198 (16.7) | 2345 (17.8) | 2859 (21.7) |
| Post-secondary non-tertiary | 4414 (11.2) | 1471 (11.2) | 1560 (11.9) | 1383 (10.5) |
| Tertiary | 25157 (63.8) | 8685 (66.0) | 8455 (64.3) | 8017 (61.0) |
| **Ethnic group - White (%)** | 38376 (97.3) | 12739 (96.9) | 12807 (97.4) | 12830 (97.5) |
| **BMI^a^ (%)** |  |  |  |  |
| Normal | 16582 (42.0) | 5284 (40.2) | 5710 (43.4) | 5588 (42.5) |
| Overweight | 14862 (37.7) | 5294 (40.2) | 4890 (37.2) | 4678 (35.6) |
| Obese | 8015 (20.3) | 2575 (19.6) | 2553 (19.4) | 2887 (21.9) |
| **Drinking daily or almost daily^a^ (%)** | 6776 (20.7) | 2354 (21.8) | 2214 (20.2) | 2208 (20.2) |
| **Smoking status^a^ (%)** |  |  |  |  |
| Never | 24613 (62.4) | 8438 (64.2) | 8212 (62.4) | 7963 (60.5) |
| Previous | 13504 (34.2) | 4300 (32.7) | 4514 (34.3) | 4690 (35.7) |
| Current | 1342 ( 3.4) | 415 ( 3.2) | 427 ( 3.2) | 500 ( 3.8) |
| **Neuroticism z-score (mean (SD))** | 0.00 (1.00) | -0.98 (0.15) | -0.22 (0.30) | 1.20 (0.67) |

N, number of participants; SD, Standard deviation; BMI, body mass index.

^a^ Measured at the imaging assessment.

## eTable 10. Association of neuroticism z-score with neuroimaging outcomes and cognitive function stratified by age at the imaging assessment

|  | **Age < 65 (N=18514) ^a^** | | **Age ≥ 65 (N=20945)** | |
| --- | --- | --- | --- | --- |
| **Neuroimaging outcomes** | **β (95% CI)** | ***p*** | **β (95% CI)** | ***p*** |
| Total brain volume | 0.00 (-0.01 to 0.02) | 0.810 | 0.00 (-0.01 to 0.01) | 0.977 |
| Total white matter | 0.01 (-0.01 to 0.03) | 0.189 | 0.01 (-0.01 to 0.02) | 0.218 |
| Total grey matter | -0.01 (-0.02 to 0.01) | 0.308 | -0.01 (-0.02 to 0.00) | 0.137 |
| Total hippocampus | 0.01 ( 0.00 to 0.03) | 0.106 | 0.00 (-0.02 to 0.01) | 0.703 |
| Left hippocampus | 0.01 (-0.01 to 0.02) | 0.474 | 0.00 (-0.02 to 0.01) | 0.913 |
| Right hippocampus | 0.02 ( 0.00 to 0.03) | 0.035 | 0.00 (-0.02 to 0.01) | 0.573 |
| White matter hyperintensities | 0.02 ( 0.01 to 0.04) | 0.009 | 0.02 ( 0.00 to 0.03) | 0.015 |
| **Cognitive function** |  |  |  |  |
| Complex processing speed | -0.05 (-0.06 to -0.03) | <0.001 | -0.06 (-0.07 to -0.04) | <0.001 |
| Verbal and numerical reasoning | -0.03 (-0.04 to -0.01) | <0.001 | -0.02 (-0.04 to -0.01) | 0.002 |
| Non-verbal reasoning | -0.04 (-0.05 to -0.02) | <0.001 | -0.03 (-0.04 to -0.01) | <0.001 |
| Working memory | -0.02 (-0.04 to -0.01) | 0.006 | -0.04 (-0.06 to -0.02) | <0.001 |
| Verbal declarative memory | -0.03 (-0.04 to -0.01) | 0.002 | -0.02 (-0.04 to -0.01) | 0.005 |
| Executive function (trail making test A) | -0.03 (-0.05 to -0.01) | <0.001 | -0.03 (-0.05 to -0.02) | <0.001 |
| Executive function (trail making test B) | -0.03 (-0.04 to -0.01) | 0.002 | -0.04 (-0.05 to -0.02) | <0.001 |

^a^ Age measured at the imaging assessment.

Imaging-related confounds (head size, head motion, head and table position, and imaging center) were regressed out from the neuroimaging outcomes (WMH volumes were log-transformed). Models were adjusted for age, sex, ethnicity, education, quintiles of the Townsend deprivation index (TDI), smoking status, alcohol consumption, body mass index (BMI). Sex, ethnicity, and TDI were measured at baseline, and for other covariates (age, education, smoking status, alcohol consumption, and BMI), measurements from the imaging study were used. CI, confidence interval.

**Reference**

1.	Lee JJ, Wedow R, Okbay A, et al. Gene discovery and polygenic prediction from a genome-wide association study of educational attainment in 1.1 million individuals. *Nature Genetics*. 2018/08/01 2018;50(8):1112-1121. doi:10.1038/s41588-018-0147-3

2.	Carter AR, Gill D, Davies NM, et al. Understanding the consequences of education inequality on cardiovascular disease: mendelian randomisation study. *BMJ*. 2019;365:l1855. doi:10.1136/bmj.l1855

3.	Foster HME, Celis-Morales CA, Nicholl BI, et al. The effect of socioeconomic deprivation on the association between an extended measurement of unhealthy lifestyle factors and health outcomes: a prospective analysis of the UK Biobank cohort. *Lancet Public Health*. Dec 2018;3(12):e576-e585. doi:10.1016/s2468-2667(18)30200-7

4.	World Health Organization. A healthy lifestyle - WHO recommendations. Updated May 2010. Accessed February 6, 2023. <https://www.who.int/europe/news-room/fact-sheets/item/a-healthy-lifestyle---who-recommendations>

5.	White IR, Royston P. Imputing missing covariate values for the Cox model. *Statistics in medicine*. 2009;28(15):1982-1998.

6.	Eekhout I, de Vet HCW, Twisk JWR, Brand JPL, de Boer MR, Heymans MW. Missing data in a multi-item instrument were best handled by multiple imputation at the item score level. *Journal of Clinical Epidemiology*. 2014/03/01/ 2014;67(3):335-342. doi:<https://doi.org/10.1016/j.jclinepi.2013.09.009>

7.	Rubin DB. *Multiple imputation for nonresponse in surveys*. vol 81. John Wiley & Sons; 2004.

8.	Ebenau JL, van der Lee SJ, Hulsman M, et al. Risk of dementia in APOE ε4 carriers is mitigated by a polygenic risk score. *Alzheimers Dement (Amst)*. 2021;13(1):e12229. doi:10.1002/dad2.12229

9.	Lange T, Vansteelandt S, Bekaert M. A simple unified approach for estimating natural direct and indirect effects. *Am J Epidemiol*. Aug 1 2012;176(3):190-5. doi:10.1093/aje/kwr525
